# Predictive value of 8-hydroxy-2′-deoxyguanosine and 8-nitroguanine production in radiation-induced skin damage after postoperative breast cancer radiotherapy

**DOI:** 10.37349/etat.2025.1002336

**Published:** 2025-09-22

**Authors:** Emmanouil K. Verigos, Sofia Sagredou, Kosmas E. Verigos, Kyriakos Orfanakos, Constantinos E. Alifieris, Maria V. Deligiorgi, Panagiotis Dalezis, Mihalis I. Panayiotidis, Petros N. Karamanakos, Dimitrios T. Trafalis

**Affiliations:** IRCCS Istituto Romagnolo per lo Studio dei Tumori (IRST) “Dino Amadori”, Italy; ^1^Department of Radiation Oncology, General Anticancer Oncology Hospital of Athens “O Agios Savvas”, 11522 Athens, Greece; ^2^Laboratory of Pharmacology, Faculty of Medicine, National and Kapodistrian University of Athens, 11527 Athens, Greece; ^3^Department of Radiation Therapy, 401 General Military Hospital, 11525 Athens, Greece; ^4^Department of Hepatobiliary and Transplant Surgery, St. Vincent’s University Hospital, D04 T6F4 Dublin, Ireland; ^5^Department of Cancer Genetics, Therapeutics & Ultrastructural Pathology, The Cyprus Institute of Neurology & Genetics, Nicosia 2371, Cyprus

**Keywords:** DNA damage, 8-hydroxy-2′-deoxyguanosine, 8-nitroguanine, radiation therapy, skin toxicity, predictive markers, breast cancer

## Abstract

**Aim:**

During radiation treatment, reactive oxygen species (ROS) and nitrogen species (RNS) are produced and, by extension, DNA adducts known as 8-hydroxy-2′-deoxyguanosine (8-OHdG) and 8-nitroguanine (8-NG), respectively. However, one of the most common side effects induced by radiotherapy is skin toxicity, which affects patients’ quality of life. In the present study, we aimed to investigate the potential predictive value of 8-OHdG and 8-NG by exploring the correlations between the alterations in the concentration levels of the two lesions and radiation-induced tissue injury upon exposure to external beam radiotherapy.

**Methods:**

For the purpose of this work, we collected blood serum samples from 33 breast cancer patients who received adjuvant radiotherapy. To conduct statistical analysis, we used: (1) linear adjustment to correlate the percent changes of 8-OHdG and 8-NG with the degree of toxicity; and (2) polynomial adaptation and exponential fitting to correlate the percent changes of 8-OHdG and 8-NG with the correlation coefficient *r* for the development of radiation dermatitis, respectively.

**Results:**

According to our findings, there is a statistically significant correlation between the alterations in the 8-OHdG and 8-NG levels and skin grade toxicity across time and varying radiation doses (*p* < 0.05).

**Conclusions:**

Both DNA lesions seem to possess a promising predictive role in radiation dermatitis, while the severity and exact grade of radiation-induced skin toxicity can be determined.

## Introduction

Radiotherapy is widely acknowledged as a critical tool in both curing and palliating cancer. During radiation exposure, the tumor mass shrinks and residual tumor cells are restrained, justifying the role of radiation therapy as a primary or adjuvant treatment to other therapeutic strategies, like surgery and chemotherapy. The major clinical outcomes of radiotherapy include: (1) the significant decrease in the local recurrence risk, (2) the increase in survival rates, and (3) the relief of suffering [1, 2].

In radiotherapy, two types of ionizing radiation (IR), X-rays and γ-rays, are commonly used [2]. Such treatment can be delivered through external-beam or internal radiation. With regard to the external-beam radiation, cancer patients receive photon beams, known as X-rays, produced by a linear accelerator. On the other hand, in internal radiation, or brachytherapy, γ-radiation sources are predominantly utilized, including radioactive sources implanted in the patient’s body. Given that the external-beam radiation possesses a high potential (4–20 MV), patients receive the administered radiation dosage in various fractions over the required time interval of the therapy. In contrast to external-beam radiation, internal radiotherapy utilizes focalized radiation with a range of 0.6 to 1 MV, thereby restricting the potential damage to surrounding normal tissues induced by radiotherapy [3, 4].

In living cells, the absorption of IR stimulates the production of both reactive oxygen species (ROS) and reactive nitrogen species (RNS). More precisely, the radiolysis of water leads to excitations and ionizations, generating free radicals and molecular byproducts, such as the superoxide anion radical (O_2_•^−^), hydroxyl radical (•OH), and hydrogen peroxide (H_2_O_2_) [5, 6]. Afterwards, these radicals attack the DNA structure, inducing breaks, base damage, and sugar destruction. However, guanine, the most vulnerable base to oxidation, is considered the most common target for ROS-induced base lesions [7, 8]. Additionally, its oxidation results in the attachment of a hydroxyl group (–OH) to the 8th position of the purine base, forming the oxidatively modified product, known as 8-hydroxy-2′-deoxyguanosine (8-OHdG) [9].

Furthermore, apart from the radiolysis of water, the exposure to the IR also leads to the activation of the inducible nitric oxide synthase (iNOS), producing RNS like the nitric oxide (•NO). Even though •NO is mainly inactive with cellular components, it reacts with O_2_•^−^, generating the peroxynitrite anion (ONOO^−^), a molecule of high reactivity that attacks cellular targets, including DNA bases [10–12]. Akin to ROS, the RNS entity, ONOO^−^, interacts with guanine, impelling nitrative lesions such as 8-nitroguanine (8-NG). However, the glycosidic bond between 8-NG and deoxyribose is significantly unstable, releasing this DNA lesion spontaneously and thus forming an apurinic site. This apurinic site pairs with adenine during DNA replication, resulting in G:C to T:A transversions [13–16].

Even though radiotherapy has been extensively used against cancer, patients usually experience side effects during or after treatment. Depending on the time of exposure, radiation’s side effects can be classified into short- and long-term. As for short-term side effects, they usually arise during treatment or within three months of therapy’s initiation and can be eliminated in a few weeks or months. They are also detected in the rapidly developing tissues, such as skin, hematopoietic tissues, and the gastrointestinal tract. On the other hand, long-term side effects appear over six months, are usually irreversible, and continue to evolve over time. Finally, this type of effect shows up in the slowly developing tissues, including the kidneys, heart, nerves, and endocrine system [17, 18].

Among the above-mentioned, skin damage is the most common side effect, with studies supporting that > 70% of patients who undergo radiotherapy display skin reactions known as radiation-induced skin reactions (RISRs) [19, 20]. Such RISRs can be classified as acute or chronic; according to their severity, these reactions are categorized into four grades based on the common toxicity criteria for adverse events of the National Cancer Institute Common Terminology Criteria for Adverse Events (NCI CTCAE version 3.0). Each grade comprises a range of symptoms. For instance, the first grade includes dry desquamation with generalized erythema, while the second grade encompasses intense erythema and uneven moist desquamation [21]. The third grade involves moist desquamation outside the skin folds, while the fourth grade includes ulcers, bleeding, and skin necrosis [22]. Finally, except for acute skin reactions, there are also chronic skin reactions, which represent the final stage of skin damage, lasting from months to years, and involve symptoms such as chronic ulcers and wounds, fibrosis, telangiectasia, secondary malignancies, and skin keratosis [23, 24].

The present study aims to explore the potential correlations between two biomarkers of DNA oxidative damage, 8-OHdG and 8-NG, and radiation-induced skin dermatitis, one of the most common side effects of radiotherapy. Additionally, other clinical parameters such as body mass index (BMI), body surface area (BSA), glomerular filtration rate (GFR), and hematocrit test (HCT) were also included. For the purpose of our work, blood serum samples were collected from breast cancer patients who had undergone postoperative complementary irradiation. Ex vivo determination of the serum 8-OHdG and 8-NG levels was performed, and their altered concentration levels were related to skin dermatitis grade. Our findings suggest a positive correlation between the alteration in their concentration levels and skin dermatitis grade, indicating the prognostic value of the two biomarkers.

## Materials and methods

### Radiotherapy patients

A total of 33 female patients with stage I to stage III breast adenocarcinoma underwent postoperative adjuvant radiotherapy (52% of patients were found at stage I, 45% at stage II, and 3% at stage III). Their ages ranged from 48 to 77 years, with a median of 57 years. The majority of patients (30) were postmenopausal, while only 3 were premenopausal. Additionally, 82% of the patients received adjuvant cytotoxic chemotherapy, and 12% of them received neoadjuvant chemotherapy. With regard to the above-mentioned clinical parameters (BSA, BMI, GFR, and HCT), the median BSA of the patients was 1.77 m^2^, the median BMI was 28.6 kg/m^2^, the median GFR was 97.7 mL/min, and the median HCT was 39.7% [25]. All treatments and blood sample collections were conducted at the Department of Radiation Therapy, 401 General Military Hospital in Athens, Greece.

The study followed the principles outlined in the European Convention on Human Rights and Biomedicine (law 2919/1998) and was approved by the Hospital Scientific Board and the Board of Bioethics of National and Kapodistrian University of Athens (approval number #053).

### Treatment

Initially, all patients underwent a computed tomography (CT) scan, which was followed by a three-dimensional (3D) conformal radiation therapy. Treatment was administered postoperatively, targeting the entire breast, axillary area, and the submandibular and subclavian lymph nodes, using opposed tangential fields with 6 MV photons. The total irradiation received by the patients ranged from 56 to 60 Gy with an additional boost of 10 Gy applied to the area of the initial disease (tumor bed). The referred radiation doses were delivered at daily doses of 2 Gy for 5 consecutive days in a week. Additionally, none of the patients underwent chemotherapy during radiation treatment.

### Sample collection and preparation

For the purpose of this study, blood samples were collected prior to the initiation of radiation therapy on day 0 (D0), after treatment, as well as on days 14 (2 weeks; D14), 28 (4 weeks; D28), and 56 [8 weeks; D56 (2 weeks after the end of radiotherapy)]. To obtain the blood samples, disposable and sterile needles were used. Once collected, all samples were transferred into vacuum tubes without an anticoagulant. All samples were centrifuged at 3,000 × *g* for 10 min, straightforward (10–15 min), and then the precipitates were removed. Following centrifugation, the supernatant serum was transferred to 1.5 mL labeled centrifuge tubes and retained at –80°C until analysis.

### ELISA measurement of plasma 8-OHdG

Serum 8-OHdG levels were estimated according to the OxiSelect^TM^ Oxidative DNA Damage ELISA kit (Catalogue number STA-320, CELL BIOLABS, Inc., USA), a competitive ELISA with a detection range of 100 pg/mL to 20 ng/mL. Following the manufacturer’s protocol, samples and standards were added to a 96-well plate pre-coated with 8-OHdG-BSA conjugate. After blocking and standard curve preparation, 50 μL of each sample or standard was incubated with anti-8-OHdG antibody, followed by the addition of a secondary antibody-enzyme conjugate. Afterwards, 100 μL of substrate solution was loaded, and samples were incubated at room temperature for 2 to 30 min. Finally, 100 μL of stop solution was added to terminate the enzymatic reaction. The absorbance was immediately measured on an ELISA reader at 450 nm (Model number 16948, Versamax, Orleans, USA). All measurements were performed in triplicate.

### ELISA measurement of plasma 8-NG

Serum 8-NG levels were measured using the OxiSelect^TM^ Nitrosative DNA/RNA Damage ELISA kit (Catalogue number STA-825-5, CELL BIOLABS, Inc., USA), a competitive ELISA with a sensitivity of 1 ng/mL. Following the manufacturer’s instructions, standards and samples were applied to a microplate pre-coated with 8-NG-BSA conjugate. After blocking, a standard curve (0–1,000 ng/mL) was prepared. Samples and standards (50 µL) were incubated with anti-8-NG antibody, followed by horseradish peroxidase (HRP)-conjugated secondary antibody. After three washing steps, 100 μL of substrate solution was applied, and the incubation time ranged from 2 to 30 min. To terminate the enzymatic reaction, 100 μL of stop solution was added, and then the absorbance was measured immediately on an ELISA reader at 450 nm (Model number 16948, Versamax, Orleans, USA). All measurements were carried out in triplicate.

### Statistical analysis

Statistical analyses were performed using Microsoft Excel (Microsoft Hellas 2019, Athens, Greece) and Origin Pro 9.0 (OriginLab Corp., Northampton, MA, USA). Pearson’s linear correlation (*r*) was used to assess the relationships between 8-OHdG and 8-NG and patients’ clinical characteristics, including BMI, age, BSA, GFR, HCT, and skin toxicity grade. The association between the percentage alterations in 8-OHdG and 8-NG levels and toxicity grade was evaluated through linear regression. Additionally, polynomial and exponential fitting were applied to explore the correlation of these changes in 8-OHdG and 8-NG levels with the development of radiation dermatitis. Statistical significance was assumed at *p <* 0.05.

## Results

### Plasma 8-OHdG levels in correlation to skin dermatitis grade

**Figure 1:**
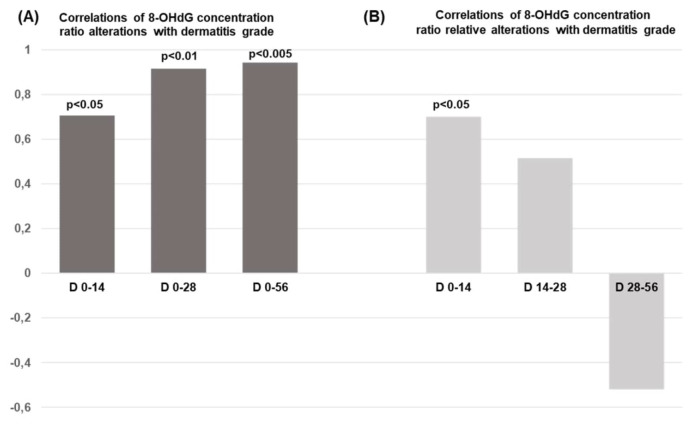
**Correlations of 8-OHdG concentration ratios alterations with dermatitis grade. (A)** Correlations of 8-OHdG ratio alterations with dermatitis grade over time (Days: D) and radiation therapy dose; **(B)** relationship between the relative alterations in 8-OHdG and the tested dermatitis grade during radiation treatment. The total therapeutic radiation dose ranged from 56 to 60 Gy. 8-OHdG: 8-hydroxy-2′-deoxyguanosine.

According to Figure 1A, the 8-OHdG alteration is significantly correlated with the skin dermatitis grade induced on D14 (*p* < 0.05), D28 (*p* < 0.01), and D56 (*p* < 0.005). Nonetheless, our data indicate the absence of any relation between 8-OHdG alteration and patients’ parameters, including BMI, BSA, GFR, and HCT [25]. It is worth mentioning that the 8-OHdG relative alterations within two weeks of radiation therapy imply the potential of predicting radiation-induced skin dermatitis with statistical significance *p* < 0.05 (Figure 1B). As our previous findings suggest, the potential alterations in the clinical parameters BMI, BSA, GFR, and HCT cannot be predicted in relation to the 8-OHdG relative alterations over time and across varying radiation doses [25].

**Figure 2:**
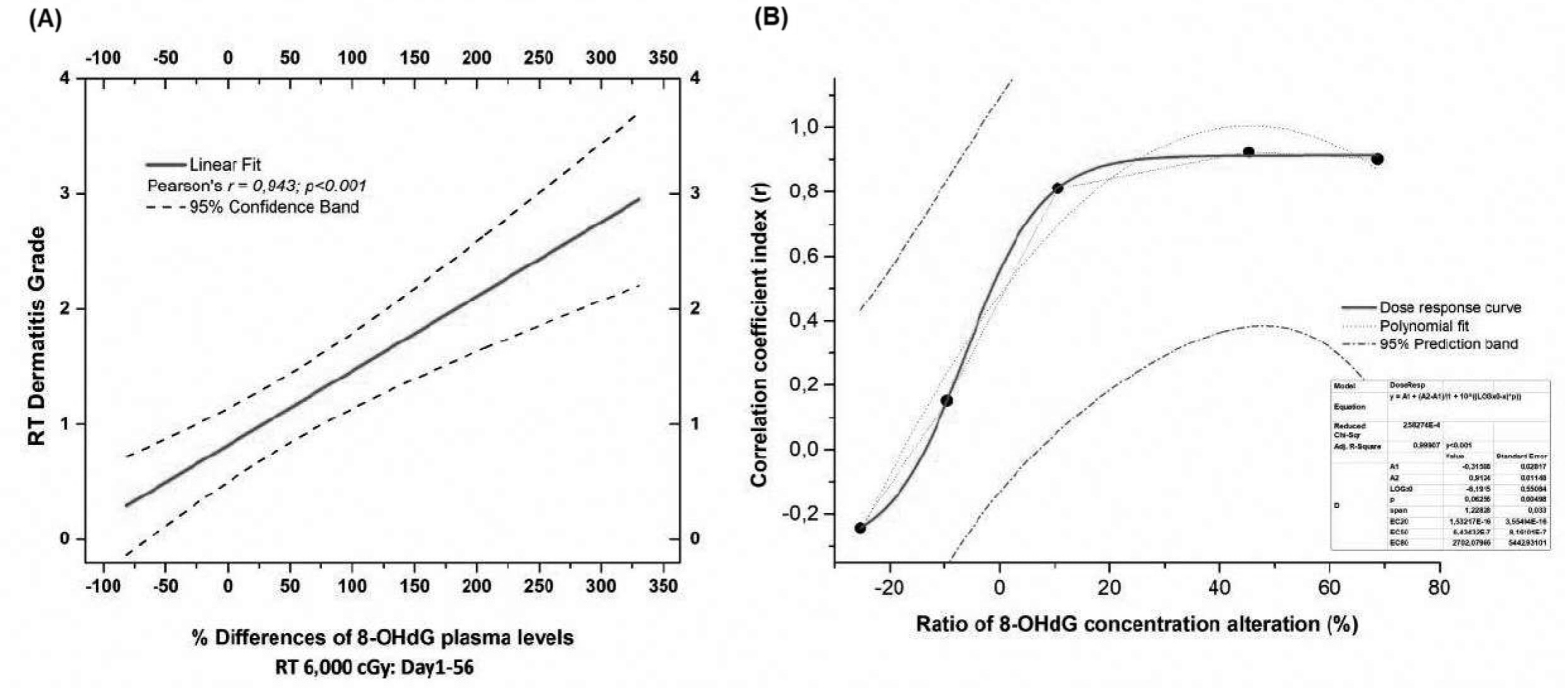
**Correlations of % 8-OHdG ratio alterations with dermatitis grade. (A)** Linear correlation of % 8-OHdG relative alterations with actinic dermatitis in patients’ plasma (Pearson’s *r* = 0.943, *p* < 0.001 and 95% confidence interval); **(B)** correlation of ratio % 8-OHdG alterations with coefficient correlation index (*r*) for the development of actinic dermatitis. Radiation doses ranged from 0 to 60 Gy. The five dots in Figure 2B represent the dosage range from 0 to 60 Gy (0, 10, 20, 40, and 60 Gy). 8-OHdG: 8-hydroxy-2′-deoxyguanosine; RT: radiation therapy.

Further data that supports the correlation between 8-OHdG and actinic dermatitis can be provided by Figures 2A and 2B. More specifically, Figure 2A displays a notable linear correlation of % 8-OHdG relative alterations with skin dermatitis grade across the different radiation doses (0–6,000 cGy) from D1 to D56. Our data suggest that the determination of % 8-OHdG levels provides the possibility of defining a patient’s skin dermatitis grade. A statistically significant correlation appears between the % 8-OHdG alterations ratios and correlation coefficient index (*r*) (Figure 2B). The model dose, which was used in statistical analysis, followed polynomial fit adaptation according to the equation $y=A1+\frac{\left(A2-A1\right)}{1+10^{\left(\log(x0\right)-x)\times p}}$, with a minimum *χ*^2^ = 2.58274 × 10^–4^ and *r*^2^ = 0.99907 (*p* < 0.001). Furthermore, Figure 2B presents a different perspective as far as concerns the correlation of 8-OHdG levels with skin dermatitis grade. According to it, it is clear that there is a linear correlation between 8-OHdG expression and radiation-induced skin damage; however, our data suggest a saturation effect. More specifically, beyond a certain point, further elevation of 8-OHdG levels does not induce further skin dermatitis severity, and thus, grade III is not exceeded. Interestingly, at the lower doses of irradiation, 10 to 15 Gy, we observed a decrease rather than an increase in baseline 8-OHdG levels, which is due to the organism’s antioxidant capacity. Further irradiation leads to cellular exhaustion and consequently to the depletion of antioxidant mechanisms, inducing an increase in 8-OHdG levels.

**Figure 3:**
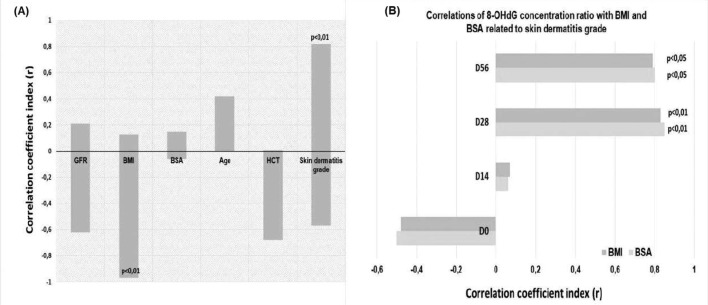
**Correlation of 8-OHdG levels with clinical parameters, including skin dermatitis grade. (A)** Correlation of 8-OHdG in terms of coefficient correlation with the clinical parameters GFR, BMI, BSA, age, HCT, and skin dermatitis grade; **(B**) correlation of the two ratios, 8-OHdG/BMI and 8-OHdG/BSA, with skin dermatitis grade in relation to the time of treatment and radiation dose. Patients received at D0 → 0 Gy, at D14 → 20 Gy, at D28 → 40 Gy, and at D56 → 60 Gy. 8-OHdG: 8-hydroxy-2′-deoxyguanosine; BMI: body mass index; BSA: body surface area; D: day; GFR: glomerular filtration rate; HCT: hematocrit test.

As Figure 3A shows, a negative correlation appears between the production of 8-OHdG and BMI, as the 8-OHdG is significantly more diluted in patients with a higher BMI than in patients with a lower BMI. Α statistically significant correlation (*p* < 0.01) has also been demonstrated between the radiation-induced 8-OHdG and skin dermatitis grade (Figure 3A). On the other hand, the production of 8-OHdG is not associated with the remaining clinical parameters, such as age, GFR, HCT, and BSA. According to Figure 3B, both ratios, 8-OHdG/BMI and 8-OHdG/BSA, are associated with skin dermatitis grade on D28 and D56, where patients received 4,000 cGy (*p* < 0.01) and 6,000 cGy (*p <* 0.05), respectively.

### Plasma 8-NG levels in correlation to skin dermatitis grade

**Figure 4:**
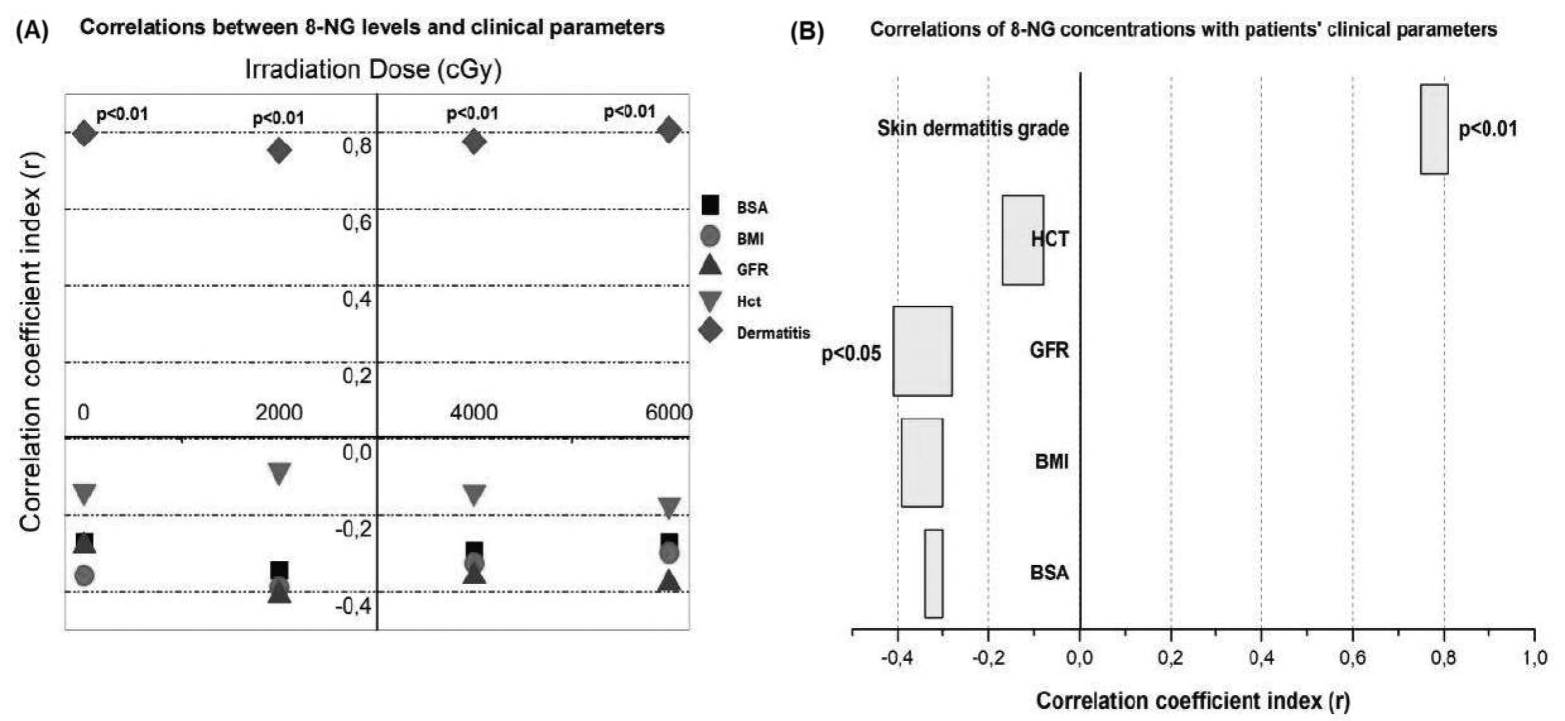
**Correlation of 8-NG levels with clinical parameters, including skin dermatitis grade. (A)** Correlation of 8-NG levels to the coefficient correlation index (*r*) with the tested clinical parameters, including BMI, BSA, GFR, HCT, and actinic dermatitis; **(B)** correlations range of 8-NG in terms of coefficient correlation index (*r*) with the tested clinical parameters (BMI, BSA, GFR, HCT, and actinic dermatitis). 8-NG: 8-nitroguanine; BMI: body mass index; BSA: body surface area; GFR: glomerular filtration rate; HCT: hematocrit test.

As far as concerns the relationship between the nitrative lesion, 8-NG, and the radiation-induced skin dermatitis, Figure 4A supports a positive correlation of the 8-NG levels with the particular side effect after the radiation dose administration at 2,000 cGy (*p* < 0.01), 4,000 cGy (*p* < 0.01), and 6,000 cGy (*p* < 0.01). Nevertheless, the production of 8-NG is not associated with the remaining clinical parameters (BMI, BSA, GFR, and HCT), neither positively nor negatively (Figure 4A). It should be mentioned that at the baseline, prior to the initiation of radiation therapy (D0), increased serum values of 8-NG were observed in some patients compared to the median. These elevated values are attributed to the prior chemotherapy received by those patients. However, these values were not associated with the development of radiation dermatitis. On the contrary, subsequent increases of 8-NG levels during treatment were positively correlated with the severity of radiation-induced skin damage. As Figure 4B points out, there is a significant positive correlation between the enhanced levels of 8-NG and the presence of radiation dermatitis (*r* = 0.78). On the other hand, the 8-NG levels are negatively related to the GFR parameter (*p* < 0.05), suggesting that the elevated GFR decreases the 8-NG levels in the plasma of patients who were exposed to radiation therapy (Figure 4B). Furthermore, there is no correlation, positive or negative, between the 8-NG production and the remaining clinical parameters (Figure 4B).

**Figure 5:**
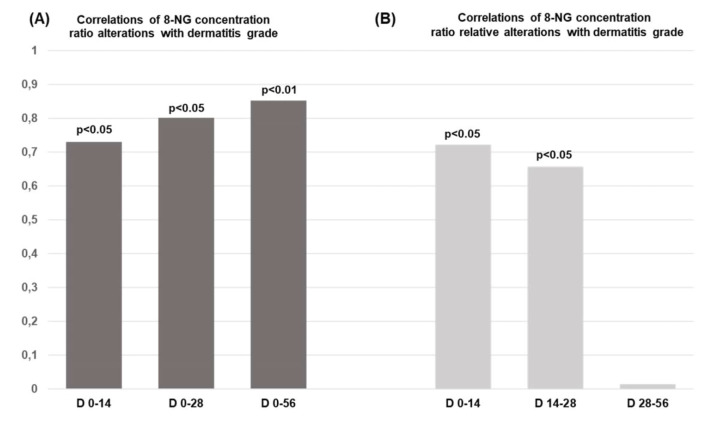
**Correlations of 8-NG concentration ratio with dermatitis grade. (A)** Correlation of the total alteration ratio of the 8-NG in terms of the coefficient correlation index (*r*) with the dermatitis grade in relation to the time and radiation dose; **(B)** correlation between the relative alterations of the 8-NG levels and the dermatitis grade by time (Days: D) and radiation doses. The radiation dosages, which were used, are the following: D0–D14 → 20 Gy, D14–D28 → 40 Gy, and D28–D56 → 60 Gy. 8-NG: 8-nitroguanine.

Furthermore, the 8-NG total alteration ratio demonstrates a statistically significant correlation with the radiation-induced skin dermatitis within the time intervals D0–D14 (*p* < 0.05), D0–D28 (*p* < 0.05), and D0–D56 (*p* < 0.01) (Figure 5A). However, according to Verigos et al. [25], there is no statistically significant correlation between the total alteration ratio of 8-NG and the above-mentioned clinical parameters, BMI, BSA, GFR, and HCT. Additionally, Figure 5B illustrates a statistically significant correlation between the relative alterations in 8-NG levels and the presence of radiation dermatitis within the time periods D0–D14 (*p* < 0.05) and D14–D28 (*p* < 0.05). The particular analysis of the intermediate time intervals of irradiation (D0–D14, D14–D28, D28–D56) points out that further production of 8-NG beyond a certain point does not influence the appearance of radiation dermatitis. Notably, the critical events appear to occur around the midpoint of therapy (D28, 40 Gy). Beyond this point, continued irradiation does not seem to exacerbate the skin damage. In contrast to radiation-induced skin dermatitis, no statistically significant correlation shows up between the relative alterations in the 8-NG levels and BMI, BSA, GFR, and HCT parameters from D14 to D28 [25]. The above-referred association may be due to radiation’s toxicity as well as to the enhanced generation of 8-NG, which belongs to radiation-induced DNA damage.

**Figure 6:**
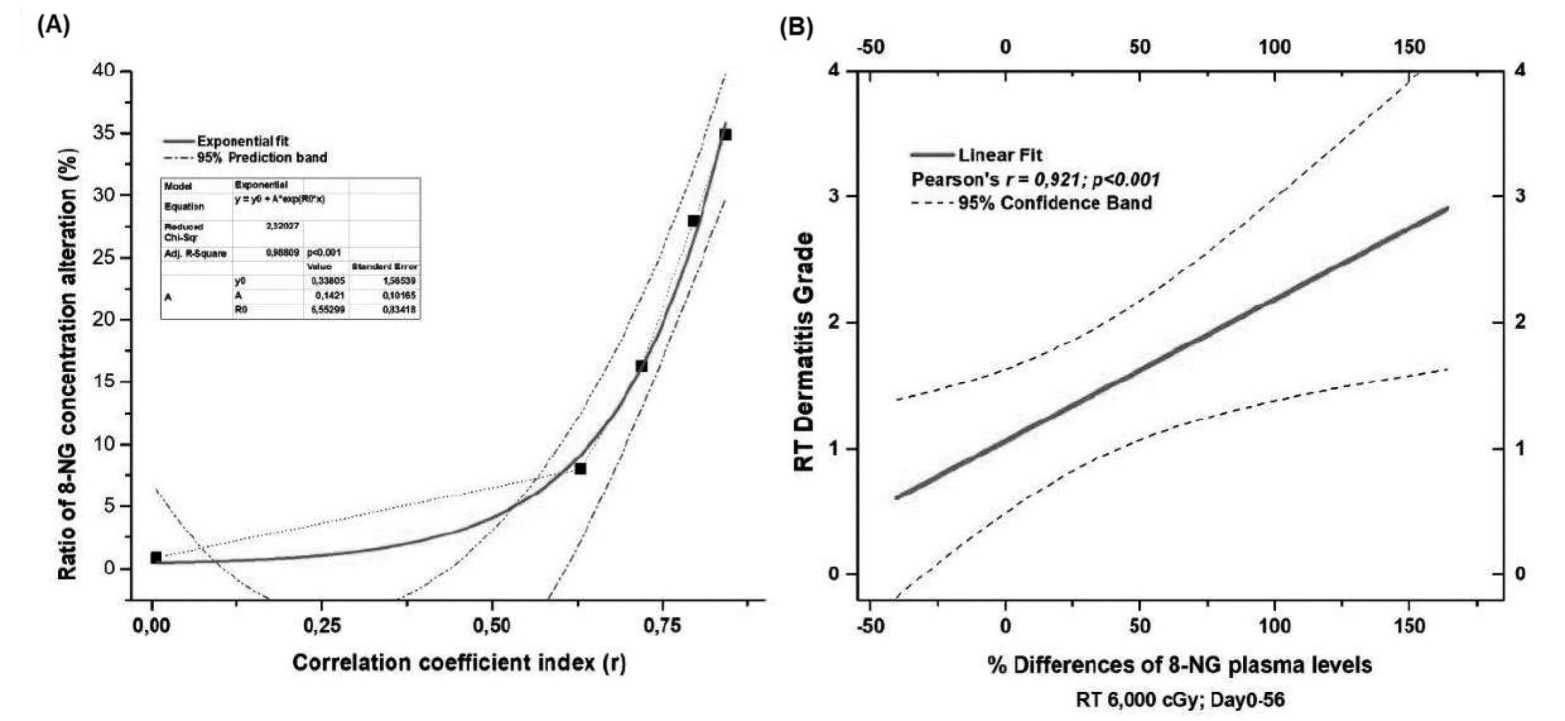
**Correlations of 8-NG concentration alterations with dermatitis grade. (A)** Correlation of 8-NG alterations ratio in terms of the coefficient correlation index (*r*) with radiation-induced skin dermatitis; **(B)** linear correlation of % 8-NG alterations with the skin dermatitis grade. The five dots in Figure 6A correspond to the doses of 0, 10, 20, 40, and 60 Gy. 8-NG: 8-nitroguanine; RT: radiation therapy.

Moreover, Figure 6A supports a correlation between the % 8-NG alterations ratio in terms of the coefficient correlation index (*r*) and radiation dermatitis. The exponential fitting mathematical model was applied according to the equation $y=y0+A\cdot e^{R0\cdot X}$, with a mixnimum *χ*^2^ = 2.32027, adjusted *χ*^2^ = 0.98809, *p* < 0.001, and 95% predictive limits. According to the results, there is a significant exponential increase in the 8-NG levels with the probability of developing radiation dermatitis, leading to the conclusion that the 8-NG levels can play a potential predictive role in the appearance of actinic dermatitis. Comparing these findings to those of Figure 2B, it is clear that different trends are illustrated: Figure 2B shows that the increase in 8-OHdG levels is early and direct, while Figure 6A demonstrates that 8-NG levels are elevated in a slower and delayed manner. This may be attributed to the enhanced renal function during the initial irradiation doses, leading this way to an enhanced excretion of 8-NG. It is likely that the production mechanism of 8-NG and the responsible enzyme, iNOS, are activated after a critical dose of radiation. As Figure 6B shows, % 8-NG alterations are linearly related to the skin dermatitis grade (Pearson’s test, *r* = 0.921, *p* < 0.001, 95% confidence limits). However, it is noteworthy that there was a significant linear relationship between % 8-NG alterations and skin dermatitis grade from D0 to D56, where radiation doses ranged from 0 to 6,000 cGy.

**Figure 7:**
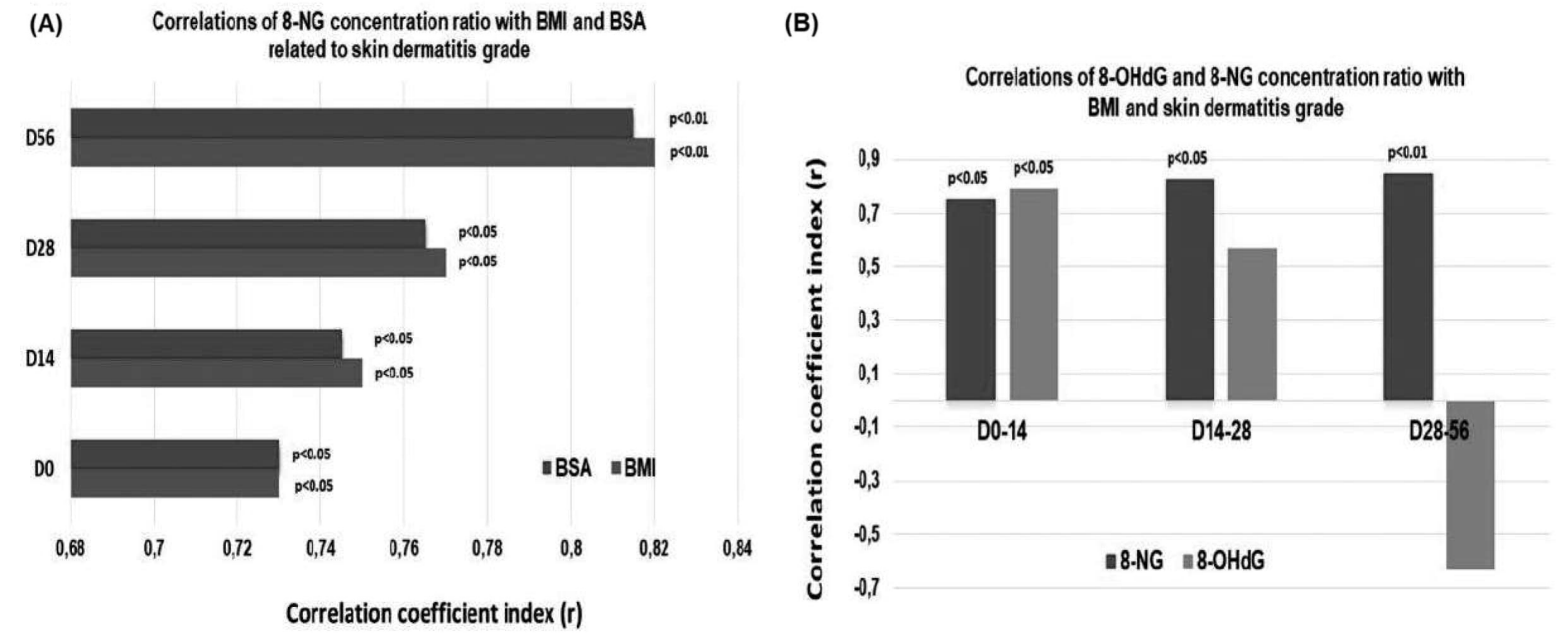
**Correlations of 8-NG/BMI and 8-NG/BSA ratios with skin dermatitis grade. (A)** Correlation of the 8-NG/BMI and 8-NG/BSA ratios with skin dermatitis grade by time and radiation dose; **(B)** correlations of the 8-NG/BMI and 8-OHdG/BMI relative alterations ratios with skin dermatitis grade. The administered radiation doses are the following: Day 0 0 Gy, Day 14 20 Gy, Day 28 40 Gy, and Day 56 60 Gy. 8-NG: 8-nitroguanine; 8-OHdG: 8-hydroxy-2′-deoxyguanosine; BMI: body mass index; BSA: body surface area.

According to Figure 7A, a statistically significant correlation shows up between the 8-NG alteration ratios and BMI on D0 and D14, where patients received 0 cGy (*p* < 0.05) and 2,000 cGy (*p* < 0.05), respectively. Similarly, 8-NG alteration ratios were significantly correlated with BMI on D28 and D56, where patients were exposed to 4,000 cGy (*p* < 0.05) and 6,000 cGy (*p* < 0.01), respectively. In addition to BMI, there is a statistically significant correlation of the 8-NG alterations ratios with BSA on D0 and D14, where the radiation dose administration was 0 cGy (*p* < 0.05) and 2,000 cGy (*p* < 0.05), respectively (Figure 7A). Additionally, Figure 7A demonstrates a statistically significant correlation between 8-NG alterations and BSA on D28 and D56 with 4,000 cGy (*p* < 0.05) and 6,000 cGy (*p* < 0.01) radiation doses being administered, respectively. It is noteworthy that there is a significant positive correlation of 8-NG alterations in terms of BMI and BSA with the development of radiation-induced skin dermatitis prior to the initiation of radiation therapy (D0) (Figure 7A).

Finally, a statistically significant correlation resulted between the 8-NG/BMI ratio and actinic dermatitis within the time intervals D0–D14 (*p* < 0.05), D14–D28 (*p* < 0.05), and D28–D56 (*p* < 0.01), where the radiation doses were 2,000, 4,000, and 6,000 cGy, respectively (Figure 7B). Furthermore, significant correlations appear between 8-OHdG/BMI and radiation dermatitis during the time periods D0–D14, where the radiation administration ranged from 0–2,000 cGy (*p* < 0.05) (Figure 7B). However, no statistically significant correlation showed up between 8-OHdG/BMI with the development of actinic dermatitis within the time intervals D14–D28 and D28–D56, where patients received 4,000 cGy and 6,000 cGy, respectively (Figure 7B). Conclusively, 8-NG, as a nitrative lesion generated by DNA damage, shows significant associations with the development of actinic dermatitis throughout the radiotherapy plan.

## Discussion

Cells are getting exposed daily to a variety of oxidizing and damaging agents originating from exogenous sources like environmental, medical, diagnostic IR, and non-IRs (X- or γ-rays, UVA radiation) as well as chemicals, and endogenous sources, including oxidative stress, predominantly produced by O_2_ metabolism, immune responses, and inflammation. In any case, the final outcome is the production of a plethora of ROS and RNS, which consist of carbonate radical (CO_3_•^−^), ONOO^−^, hypochlorous acid (HOCl), H_2_O_2_, O_2_•^−^, and •OH. Both ROS and RNS interact with cellular components, leading to the damage of DNA, lipids, and proteins. In the case of DNA, ROS and RNS attack the guanine base, inducing the formation of 8-OHdG and 8-NG, which serve as established markers of oxidative and nitrative DNA damage, respectively. It is noteworthy that measuring oxidative DNA lesions like 8-OHdG and 8-NG is not without controversy [26–29]. According to Cooke et al. [30], several methodological limitations exist, including the lack of standardization across detection techniques (e.g., ELISA), variability in sample preparation and storage elevating the lesion levels, potential cross-reactivity or nonspecificity in immunoassays, and challenges in distinguishing endogenous damage from ex vivo oxidation during sample preparation. As a consequence, these restrictions have led to inconsistent findings across the literature regarding the 8-OHdG and 8-NG markers; thus, their interpretation should be context-dependent and supported by methodological rigor and appropriate controls [31, 32].

Except for 8-OHdG and 8-NG, other forms of DNA lesions are single-strand breaks and oxidized bases like 8-oxo-7,8-dihydro-2-deoxyguanosine (8-oxodG) and 5,6-dihydroxy-5,6-dihydrothymine (Tg). These types of DNA damage are primarily repaired by the base excision repair (BER) pathway and, to a lesser extent, by the nucleotide excision repair (NER) pathway. Nevertheless, cells may bypass DNA damage using specific DNA polymerases and enter DNA replication, causing this way mutations and chromosomal lesions. Alternatively, extensive unrepaired DNA damage can trigger the apoptotic pathway. In the case of inadequate repair and persistent DNA insult, accumulated mutations and genomic instability create a pre-cancerous state that may ultimately progress to malignancy [32].

Even though endogenous oxidative DNA damage is a baseline physiological process, it can be remarkably enhanced under stress conditions such as those of irradiation, inflammation, hypoxia, and metabolic dysregulation. In the context of radiotherapy, IR leads to oxidative stress through water radiolysis and activation of endogenous enzymatic sources, including nicotinamide adenine dinucleotide phosphate (NADPH) oxidases, mitochondrial respiration, and NOS. IR-induced DNA damage can work as a mechanism of cytotoxicity, while in the meantime, it may trigger inflammatory cascades, contributing to tissue injury, like radiation dermatitis [33, 34].

With regard to radiation-induced dermatitis (RID), it is considered one of the most prevalent side effects of radiotherapy [35–37]. When a patient receives external beam radiation therapy, IR inevitably penetrates the patient’s skin, triggering an inflammatory response. As far as concerns breast cancer patients, over 70% of them undergo adjuvant radiotherapy upon lumpectomy or radical mastectomy. It is also estimated that approximately 90% of breast cancer patients exhibit at least one symptom of faint erythema (CTCAE = 1), which appears as a rash and causes sensations of burning, itching, and discomfort to the patients. For over 30% of them, erythema can evolve into a severe and moist desquamation, raising, therefore, the infection risk. However, there are extreme cases in which the course of treatment can be prevented due to the severity of erythema, jeopardizing the patient’s therapeutic future. Moist desquamation usually appears a few weeks after the end of radiotherapy and is strongly related to long-term fibrosis and permanent skin damage, leading to a decreased quality of life [38, 39].

With regard to potential mechanisms, RISRs’ development is strongly linked to inflammatory response and oxidative stress. Cytokines (IL-1, IL-3, IL-5, IL-6, and TNF-α), chemokines (eotaxin and IL-8), receptor tyrosine kinases, and adhesion molecules trigger a local inflammatory response leading to ongoing tissue damage and loss of protective barrier [40]. Alternatively, IR-generated free radicals upregulate subtypes of NADPH oxidase, such as NADPH oxidase 1 (NOX-1), dual oxidase 1 (DUOX1), and DUOX2, which are highly stable and continuously produce ROS following exposure. ROS production sustains molecular changes, destroys DNA, lipids, and proteins, stimulates early-response transcription factors and signal transduction pathways, leading to skin tissue damage [41–43].

Factors that are usually implicated in radiation-induced skin toxicity are: (1) patient-related factors including genetic susceptibility, age, nutritional status, smoking, existing skin conditions, and BMI; (2) radiation’s treatment conditions such as radiation dose, fractionation, delivery techniques, location, and size of the treatment area; and (3) whether the patient receives concurrent chemotherapy, which deteriorates radiotherapy’s effects. Given that total radiation dose, dose per fraction, and dose volume on the exposed surfaces affect the risk of radiation dermatitis, it is clear that skin toxicity is a dose-dependent side effect of radiotherapy [39, 44]. For instance, when a patient receives external beam radiotherapy, erythema appears at skin doses ≥ 6 Gy, dry desquamation at skin doses ≥ 20 Gy, and moist desquamation at skin doses ≥ 30 Gy [45].

Beyond a patient’s clinical picture, there are several patient-related factors that constitute suitable markers for evaluating individual radiosensitivity. Examples include γH2AX and TP53; previous findings have highlighted a decrease in γH2AX levels and activation of TP53 upon radiation treatment, pointing them out as indicators of DNA damage response and radiosensitivity [46, 47]. IL-6 and IL-1β are commonly used in clinical practice as predictive biomarkers as well. Nonetheless, there is a growing trend toward the era of personalized cancer treatment and risk assessment. Of particular importance is the discovery of biomarkers linked to normal tissue radiosensitivity and toxicity in easily accessible samples such as blood (serum, blood cells, and plasma). Such molecules, protein or not, are considered promising biomarkers that may open new avenues in personalized medicine [48].

As previously described, ROS and RNS production are known consequences during radiotherapy, and 8-OHdG and 8-NG are considered established markers of oxidative and nitrative stress, respectively. Interestingly, Verigos et al. [25] showed a strong dose-dependent relationship between the production of the two biomarkers, 8-OHdG and 8-NG, and radiotherapy conditions. Once normal tissue is irradiated, 8-OHdG and 8-NG are produced, while the alterations in their concentration levels are positively correlated with the radiation dose and time of exposure. These findings prompted us to investigate the relationship between the concentration levels of the two DNA adducts, 8-OHdG and 8-NG, and the development of radiation-induced skin injury in breast cancer patients receiving adjuvant radiotherapy. Our findings indicate a statistically significant correlation between the percent alterations in these biomarkers and the grade of skin toxicity over time and across varying radiation doses (*p* < 0.05).

So far, the literature provides hardly any findings on the potential predictive value of 8-OHdG or 8-NG for RID. However, our data support the promising predictive role of 8-OHdG as its alteration levels are positively correlated with skin toxicity grade throughout the radiation therapy regimen (Figures 1–3) (*p* < 0.01). It is noteworthy that among the tested clinical parameters, the production of 8-OHdG was negatively correlated with BMI, as expected, since the biomarker was more diluted in patients with a higher BMI than in patients with a lower BMI (Figure 3). Previous studies have shown that a higher BMI is positively associated with increased levels of oxidative stress. More specifically, as BMI increases, so do the markers of oxidative stress in the organism. An interplay also appears between oxidative stress and obesity. In other words, oxidative stress is not only a consequence but also stimulates obesity. The increased intake of fats, carbohydrates, and saturated fatty acids, especially those of trans-fatty acids, leads to enhanced oxidative stress through biochemical pathways, including the synthesis of superoxide anion via oxidative phosphorylation, glyceraldehyde auto-oxidation, protein kinase C (PCK) activation, and polyol and hexosamine pathways [49–52]. Studies have also shown that the levels of antioxidant enzymes, such as Cu-Zn superoxide dismutase (SOD) and glutathione peroxidase (GPx), are lower in the erythrocytes of obese subjects compared to those of nonobese individuals [53, 54]. Another way through which obesity enhances oxidative stress is through the secretion of proinflammatory cytokines. Adipose tissue excretes the proinflammatory cytokines TNF-α, IL-1β, and IL-6, triggering ROS production. On the other hand, oxidative stress triggers the deposition of adipose tissue, which involves preadipocyte proliferation, adipocyte differentiation, and growth, leading this way to obesity [55]. It is noteworthy that the BMI factor is of utmost importance as it can be useful as a predictive marker for acute skin toxicity in breast cancer patients. Studies have shown that the size of the mammary gland is highly related to the risk of actinic dermatitis, making it necessary to accurately measure the breasts of large size so as to identify patients with a high risk of skin toxicity [56].

Akin to 8-OHdG, 8-NG has also emerged as a promising candidate predictive marker for radiation dermatitis, as its alteration levels were positively correlated with radiation dermatitis grade over time and across varying radiation doses (Figures 4–7) (*p* < 0.01). Nonetheless, it is noteworthy that the absence of a statistically significant correlation between the alterations in 8-NG levels and radiation dermatitis prior to radiotherapy points out its promising predictive value (Figure 4). It can be assumed that the enhanced production of 8-NG found in the blood serum of patients may be due to the prior administration of adjuvant chemotherapy. As is known, several antineoplastic drugs stimulate the production of ROS and RNS, destroying tumors and biological systems. Such agents are: anthracyclines, alkylating agents, platinum complexes, epipodophyllotoxins, and camptothecins [57–60]. For instance, doxorubicin activates the NF-κB pathway, which in turn triggers iNOS, leading by extension to increased NO production [61]. Moreover, in 3D breast cancer spheroids, taxanes such as docetaxel regulate the enzymatic activity of both iNOS and endothelial NOS (eNOS), elevating NO levels, though to a lesser extent than doxorubicin [62]. Similarly, cisplatin upregulated iNOS expression, while eNOS and neuronal NOS (nNOS) expression levels were reduced in OV2008 ovarian cells only [63]. Finally, treatment with cyclophosphamide resulted in increased iNOS expression levels in bone marrow and blood cells, contributing to enhanced NO levels [64]. Interestingly, a negative correlation appears between GFR and 8-NG levels; increased GFR is related to reduced 8-NG levels at the initial doses of irradiation. The better the renal function, the lower the concentration of NO derivatives, probably due to enhanced renal excretion. According to our findings, a significant negative correlation was observed between alterations in 8-NG levels and GFR at the low doses of 20 Gy, which was opposite to the trend observed for 8-OHdG levels. Verigos et al. [25] have also reported this negative correlation between GFR and 8-NG levels within the initial time interval of irradiation (D0–D14). A potential mechanism behind this is the adequate renal clearance of nitrated nucleoside derivatives. A higher GFR may facilitate the excretion of 8-NG, while a reduced renal function will lead to its accumulation in the organism’s circulation. However, this relationship may be altered by the radiation-induced iNOS activation and impaired antioxidant defense mechanisms [65].

Although skin toxicity is a very common side effect in breast cancer patients, affecting up to 90% of them, it remains challenging to classify and predict it in advance. So far, there are a few ways to predict such toxicities, including clinical examination, visual inspection, and patient-related symptoms. Physiological changes related to RID, like inflammation, may increase body-surface temperature, which in turn can be detected by thermal imaging. The ability to predict such toxicities at the early stages of the treatment would enable clinicians to intervene and adjust the treatment plan accordingly [39].

Predictive models for radiation-induced toxicities can be alternatively developed with the assistance of machine learning (ML) technology. To the best of our knowledge, there are only two studies that attempted to use ML-based models in order to predict radiation-induced breast skin toxicity [66, 67]. For instance, Saednia et al. [66] identified quantitative thermal imaging biomarkers and incorporated them in ML frameworks to develop a predictive model for skin damage induced by radiotherapy. On this path, the integration of the two biomarkers, 8-OHdG and 8-NG, into ML models may enhance the accuracy in the prediction of radiation-induced skin toxicity and thus support personalized radiotherapy strategies. Therefore, the current research provides essential biological input for the development of future algorithms, and thus, the potential predictive value of 8-OHdG and 8-NG may significantly contribute to the development of future AI-driven applications.

Nevertheless, this study is not without limitations. Firstly, the relatively small sample size of 33 patients may restrict the generalizability of our findings. Secondly, our study only focused on the serum levels of 8-OHdG and 8-NG; other potential biomarkers or confounding factors, including genetic predispositions or comorbidities, were not assessed. Further studies need to be carried out with larger, more diverse cohorts and comprehensive analyses of the aforementioned factors to better validate these results and refine the predictive models.

Still, these findings have practical implications for patient management. Monitoring the levels of 8-OHdG and 8-NG during radiotherapy renders feasible the early identification of patients with a high risk of developing severe radiation dermatitis, allowing for timely intervention. Such could involve adjustments to treatment protocols, like dose fractionation, or the application of prophylactic measures, such as topical treatments to mitigate skin damage and improve patient quality of life [68–70].

The classification and prediction of skin toxicity outcomes are pretty challenging prior to radiotherapy due to the complex interplay between patient-related factors and treatment specifics. So far, BMI has been extensively used for the prediction of radiation dermatitis in breast cancer patients; however, there is still an urgent need to discover biomarkers through which skin toxicity can be predicted with efficiency and accuracy. Actinic dermatitis, the most common side effect of radiation treatment, can result from either an inflammatory response or oxidative stress, and it is related to radiation therapy in a dose-dependent manner. Based on our previous findings, both DNA lesions, 8-OHdG and 8-NG, corollaries of ROS and RNS induced upon exposure to IR, are generated in relation to radiation dose, specifying a clear dose-response relationship. The current study brings out the promising predictive value of the two biomarkers not only in terms of skin damage but also in terms of the exact grade of skin toxicity, though further studies will be carried out in that field.

## Abbreviations

3D: three-dimensional
8-NG: 8-nitroguanine
8-OHdG: 8-hydroxy-2′-deoxyguanosine
BMI: body mass index
BSA: body surface area
D: day
DUOX1: dual oxidase 1
eNOS: endothelial nitric oxide synthase
GFR: glomerular filtration rate
H_2_O_2_: hydrogen peroxide
HCT: hematocrit test
iNOS: inducible nitric oxide synthase
IR: ionizing radiation
ML: machine learning
NADPH: nicotinamide adenine dinucleotide phosphate
CTCAE: Common Terminology Criteria for Adverse Events
O_2_•^−^: superoxide anion radical
ONOO^−^: peroxynitrite anion
RID: radiation-induced dermatitis
RISRs: radiation-induced skin reactions
RNS: reactive nitrogen species
ROS: reactive oxygen species
•NO: nitric oxide
•OH: hydroxyl radical

## Declarations

### Availability of data and materials

The data analyzed in this study were obtained from the doctoral thesis of Dr. Kosmas E. Verigos. Requests for access to these datasets should be addressed to Dr. Verigos at kosmas.verigos@usa.net.

### Copyright

© The Author(s) 2025.
